# Placental Metabolomics for Assessment of Sex-specific Differences in Fetal Development During Normal Gestation

**DOI:** 10.1038/s41598-020-66222-3

**Published:** 2020-06-10

**Authors:** Michelle Saoi, Katherine M. Kennedy, Wajiha Gohir, Deborah M. Sloboda, Philip Britz-McKibbin

**Affiliations:** 1 Department of Chemistry and Chemical Biology, McMaster University, Hamilton, Canada; 2 Department of Biochemistry and Biomedical Sciences, McMaster University, Hamilton, Canada; 3 Department of Pediatrics and Obstetrics and Gynecology, McMaster University, Hamilton, Canada; 4 Farncombe Family Digestive Health Research Institute, McMaster University, Hamilton, Canada

**Keywords:** Metabolomics, Bioanalytical chemistry

## Abstract

The placenta is a metabolically active interfacial organ that plays crucial roles in fetal nutrient delivery, gas exchange and waste removal reflecting dynamic maternal and fetal interactions during gestation. There is growing evidence that the sex of the placenta influences fetal responses to external stimuli *in utero*, such as changes in maternal nutrition and exposure to environmental stressors. However, the exact biochemical mechanisms associated with sex-specific metabolic adaptations during pregnancy and its link to placental function and fetal development remain poorly understood. Herein, multisegment injection-capillary electrophoresis-mass spectrometry is used as a high throughput metabolomics platform to characterize lyophilized placental tissue (~2 mg dried weight) from C57BL/6J mice fed a standardized diet. Over 130 authentic metabolites were consistently measured from placental extracts when using a nontargeted metabolomics workflow with stringent quality control and robust batch correction. Our work revealed distinct metabolic phenotype differences that exist between male (*n* = 14) and female (*n* = 14) placentae collected at embryonic day E18.5. Intracellular metabolites associated with fatty acid oxidation and purine degradation were found to be elevated in females as compared to male placentae (*p* < 0.05, effect size >0.40), including uric acid, valerylcarnitine, hexanoylcarnitine, and 3-hydroxyhexanolycarnitine. This murine model sheds new insights into sex-specific differences in placental mitochondrial function and protective mechanisms against deleterious oxidative stress that may impact fetal growth and birth outcomes later in life.

## Introduction

The placenta is an interfacial organ that regulates complex maternal-fetal interactions during the course of pregnancy^1^. This metabolically active organ plays crucial roles in transferring nutrients and oxygen from maternal circulation to the fetus, as well as facilitating removal of waste products, and synthesizing hormones, cytokines and growth factors to promote growth, cellular regulation and immune protection for the fetus. Since the placenta is often the first organ to develop during embryogenesis in mammals, it contributes to sex-specific differences in health and disease early in development^2,3^. Normal placental function is critical to ensure optimal birth outcomes for offspring while preventing placental-induced pregnancy complications, such as intrauterine growth restriction and preeclampsia^4^. There is growing evidence demonstrating the importance of sex-specific embryonic and fetal adaptations to adverse environments *in utero*, which are largely mediated by placental genes, proteins and steroid pathways^5^. Sex-specific differences in the expression of genes encoding a number of endocrine and immune related pathways have been shown in many species including humans, and may be one mechanism regulating adaptive responses to stressors in a sex-dependent manner^6–8^. Indeed, females more readily respond to abrupt changes to intrauterine environment, where developmental adaptations ultimately lead to functional changes in placental growth and development resulting in decreased fetal growth^5^. In contrast, male feto-placental units adopt a more “minimalist approach” where they undergo very few placental changes and continue to grow normally under adverse *in utero* environment. However, this adaptation places male fetuses at higher risk to exposures that may contribute to deleterious health impacts on later development due to environmental toxins and/or sub-optimal maternal nutrition^9–11^.

Despite these known sex-specific mechanisms in fetal development, few studies have examined the impact of sexual dimorphism on placental growth, development and function in the absence of disease or adverse stimuli. Most reports have focused primarily on measuring differences in sex chromosomes and epimutations associated with placental function^12^, with sparse work aimed at characterizing the placental metabolome^13^. Metabolomics offers a nontargeted approach for the detection and identification of low molecular weight metabolites (<1 kDa) comprising a biofluid, cell, tissue or organism. Due to its sensitivity to changes in environmental and physiological stimuli, metabolomics provides a link between biochemical mechanisms and molecular phenotype that is closely associated with clinical outcomes^14^. To date, adaptive metabolic changes within placentae during pregnancy have been analyzed in the context of adverse *in utero* environments, such as pregnancy complications (*e.g*., preeclampsia, hypoxia, gestational diabetes) and maternal obesity^13,15–20^. Herein, we investigated the impact of sex on the placental metabolome in normal uncomplicated murine pregnancies using multisegment injection-capillary electrophoresis-mass spectrometry (MSI-CE-MS). This method offers a high throughput platform and accelerated data workflow for biomarker discovery in metabolomics with stringent quality control (QC) that is optimal for analysis of volume and mass-limited biospecimens^21–23^, such as lyophilized murine placental tissue (<2 mg). Our previous study revealed perturbations in murine placental metabolism elicited by a high fat maternal diet that corresponded to changes in maternal gut microbiota as well as gut barrier integrity with increased hypoxia and inflammatory markers likely impacting fetal gut development^24^; however, there were modest effects of biological sex on placental metabolism. In this work, we sought to identify unique metabolite signatures distinguishing female from male placentae during normal pregnancy following nontargeted characterization of the murine placental metabolome.

## Results

### Characterization of the murine placental metabolome

**Figure 1:**
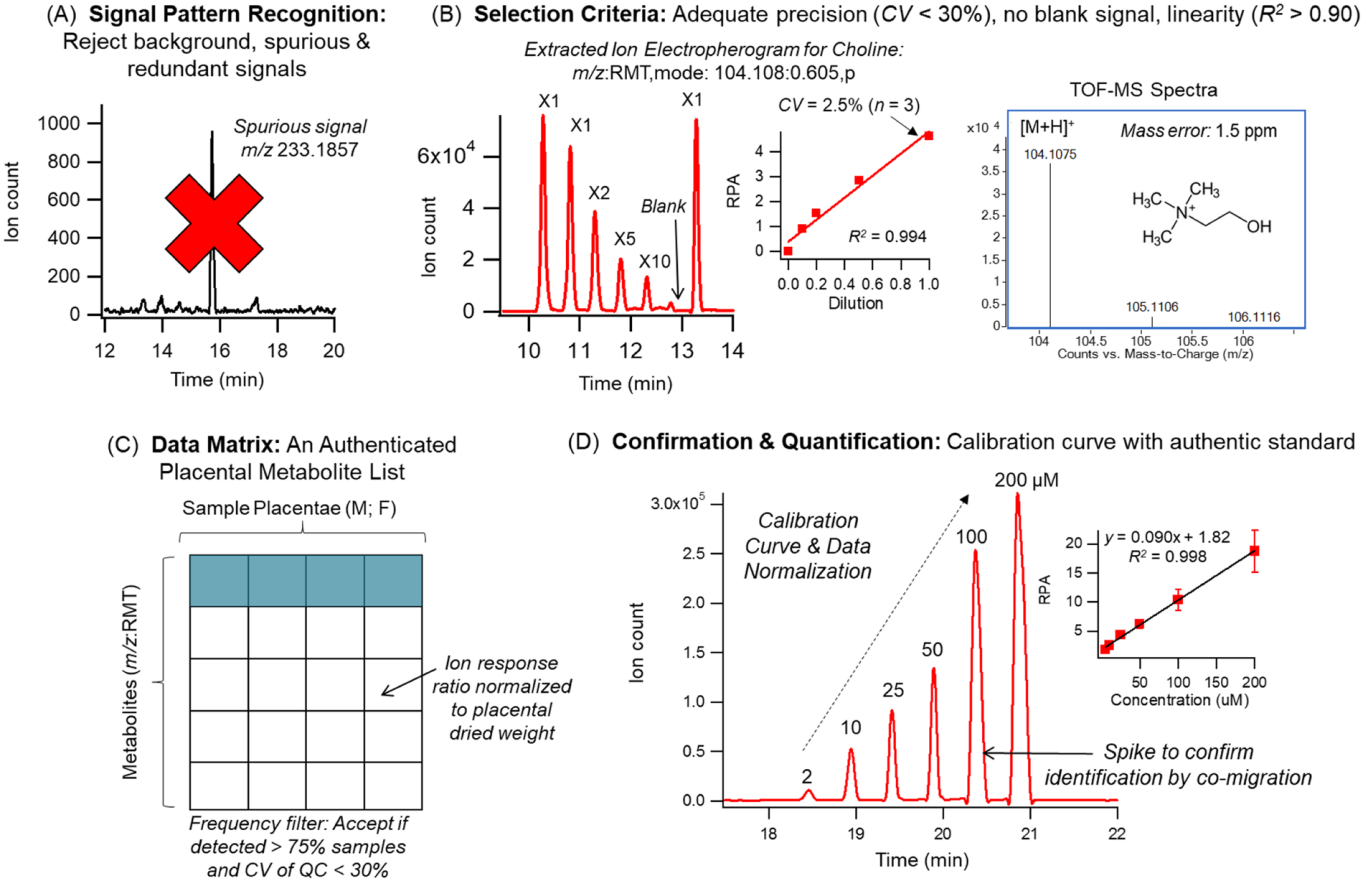
Overview of the metabolomics data workflow to authenticate placenta-derived metabolites when using multiplexed separations based on MSI-CE-MS. (**A**) A dilution trend filter was initially used to filter out background, spurious and redundant signals generated in ESI-MS to avoid false discoveries. A representative extracted ion electropherogram of a spurious peak (*m/z* 233.1857) under positive ion mode is depicted that does not fulfill selection criteria and thus removed from a list of molecular features from a pooled placental extract. (**B**) Choline (*m/z*:RMT 104.108:0.605:p) is an authentic metabolite from placenta that is measured with good precision (*CV* = 2.5%, *n* = 3), lacks of signal in the blank extract, and shows good linearity upon dilution (*R*^*2*^ > 0.90) forming a distinctive temporal signal pattern reflecting serial sample injection. (**C**) Using these selection criteria, a final data matrix of authentic placental metabolites was reliably detected in a majority of samples in the cohort (>75%) with adequate precision (*CV* < 30%) based on pooled placental extracts used as QCs (*n* = 12) in the study. (**D**) Absolute quantification for a majority of placental metabolites was performed using MSI-CE-MS where a 6-point calibration curve was performed in one experimental run; all signals were normalized to an internal standard and placental dried weight, and metabolite identification was confirmed by co-migration after spiking a calibrant standard into a pooled sample.

Comprehensive metabolite profiling of murine placental extracts was performed using a multiplexed electrophoretic separation platform applicable to the analysis of a diverse range of polar/non-polar ionic metabolites using small amounts of lyophilized tissue specimens. Figure 1 depicts an overview of the data workflow used for authenticating metabolites from unknown molecular features detected from placental tissue collected from C57BL/6J mice (*n* = 14); this process takes advantage of a serial injection format comprising of 7 samples within a single run in conjunction with temporal signal pattern recognition when using MSI-CE-MS^25^. Firstly, a dilution trend filter was used as a rigorous approach to reject background, spurious and redundant ion signals (*i.e*., in-source fragments and/or adducts, isotope peaks) generated in ESI-MS^26^ that contribute to data over-fitting and false discoveries in metabolomics as shown in Fig. 1(A). Stringent selection criteria was also applied to confirm that metabolites measured from a pooled placental extract have adequate precision (*CV* < 30%, *n* = 3) and linearity (*R*^2^ > 0.900 upon serial sample dilution) without a signal detected in a blank extract as a control. Figure 1(B) illustrates an extracted ion electropherogram for choline, a highly abundant intracellular metabolite detected from murine placental extracts satisfying all 3 selection criteria outlined above. Also, high resolution MS spectra were acquired for all metabolites that provides information on their accurate mass, charge state, and isotope pattern for determination of their most likely molecular formulae with low mass error (<5 ppm). Overall, 135 authentic and reliably measured placental metabolites (79 cations, 27 anions, 29 acidic lipids) were confirmed from over 700 molecular features initially detected (Fig. 1C). A summary of all authenticated placental metabolites are listed in Tables S1 and S2, where each metabolite is annotated based on their characteristic accurate mass, and relative migration time (*m/z*:RMT) under positive (p) or negative (n) ion mode detection. However, 122 metabolites were included in the final metabolomics data matrix as these were consistently detected in a majority (>75%) of individual female and male murine placental samples analyzed in this study (*n* = 28) from 14 murine pregnancies. Overall, placental metabolites were comprised of a wide array of compound classes associated with amino acid metabolism, redox homeostasis, central energy metabolism, and fatty acid metabolism. Most placental metabolites were confidently identified (level 1) based on mass spectral matching and co-migration when spiked with an authentic standard, whereas 12 metabolites (~10%) were putatively identified (level 2) based on comparing their MS/MS spectra to public databases. Also, 11 unknown metabolites (~10%) were detected and annotated based on their most probable molecular formula (level 3) in accordance with recommendations from the Metabolomics Standards Initiative^27^. Absolute quantification of a majority of placental metabolites was achieved via a 6-point calibration curve when using authentic standards over a 100-fold linear dynamic range as depicted in Fig. 1(D), where ion responses were normalized to a single non-deuterated internal standard (Cl-Tyr or NMS). Tables S1 and S2 in the Supplemental Information also summarize the average intracellular concentrations for placental metabolites measured in this study that were normalized to total dried weight (mmol/mg).

### High throughput metabolite profiling of placental tissue

**Figure 2:**
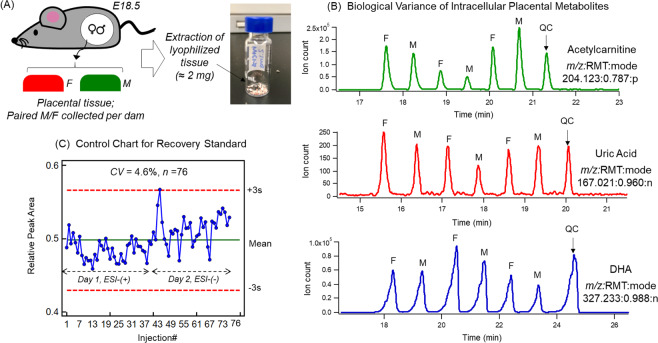
(**A**) Schematic overview of the study design where female mice were fed a standardized diet for six weeks prior to mating and at embryonic day E18.5 male and female placental tissue were collected. (**B**) Representative extracted ion electropherograms for acetylcarnitine, uric acid, and DHA that depict a 7-sample plug serial injection format used for comprehensive metabolite profiling of placental extracts by MSI-CE-MS that was run under three configurations for polar cationic, polar anionic, as well as anionic lipids. Within one experimental run, three sex-paired placental tissue extracts were simultaneously analyzed from dams together with a pooled sample as QC to assess and correct for long-term signal drift in ESI-MS. (**C**) Control charts for the recovery standard, F-Phe which was added in every tissue extract to confirm acceptable long-term technical precision (*CV* = 4.6%, *n* = 76) over two days when using MSI-CE-MS under positive and negative ion mode detection.

After characterizing the murine placental metabolome using stringent selection criteria to authenticate metabolites, MSI-CE-MS was then used to analyze individual sex-paired placental extracts (*i.e*., female and male) as shown in Fig. 2(A). In this case, between-sex differences in metabolite expression within placenta were measured by comparing their normalized ion responses by MSI-CE-MS while analyzing a QC for assessing technical precision and long-term signal drift. Figure 2(B) shows an extracted ion electropherogram for placenta derived acetylcarnitine (204.1230:0.787:p), an important mediator of fatty acid metabolism in the mitochondria. Similarly, intracellular anionic metabolites from placental extracts were also measured in this work under negative ion mode detection, including polar/hydrophilic metabolites, such as uric acid (167.0211:0.960:n), and non-polar lipids using non-aqueous buffer conditions^28^, such as the omega-3 fatty acid, docosahexaenoic acid or DHA (327.2330:0.988:n).

Thus, each murine placental extract was analyzed by three different instrumental configurations when using MSI-CE-MS. After data acquisition, instrumental performance was carefully assessed prior to data processing and statistical analysis. Two analytical batches of placental tissue were collected and analyzed over a period of two years. The first batch was analyzed in 2016 (9 pregnant mice), and the second batch was subsequently analyzed in 2018 (5 pregnant mice) using the same instrumental platform and extraction protocol in order to further increase study power. Importantly, aliquots of the same QC from the first batch of samples were analyzed in every run across both sample batches when using MSI-CE-MS. Figure 2(C) depicts a control chart for the second analytical batch analyzed demonstrating that excellent technical precision was obtained (*CV* = 4.6%, *n* = 76) over two consecutive days of analysis based on the normalized ion responses for a recovery standard (F-Phe) added to all placental extracts with only one outlier exceeding warning limits (±3 s). However, between-batch effects were nonetheless evident due to long-term signal drift (*CV* > 20%) for certain metabolites as highlighted in Fig. S1(a). To correct for system drift in ESI-MS that was compound dependent, a batch-correction algorithm based on an empirical Bayesian framework was applied when relying on QC samples analyzed in each serial injection run by MSI-CE-MS^21^. Control charts highlight considerable improvements in overall technical precision following a QC-based batch correction algorithm as shown in Fig. S1(b). Importantly, Fig. S1(c,d) demonstrate that batch adjustment did not impact the underlying data structure and natural biological variance of the murine placental metabolome.

### Sex-specific metabolic adaptations in the placenta

**Figure 3:**
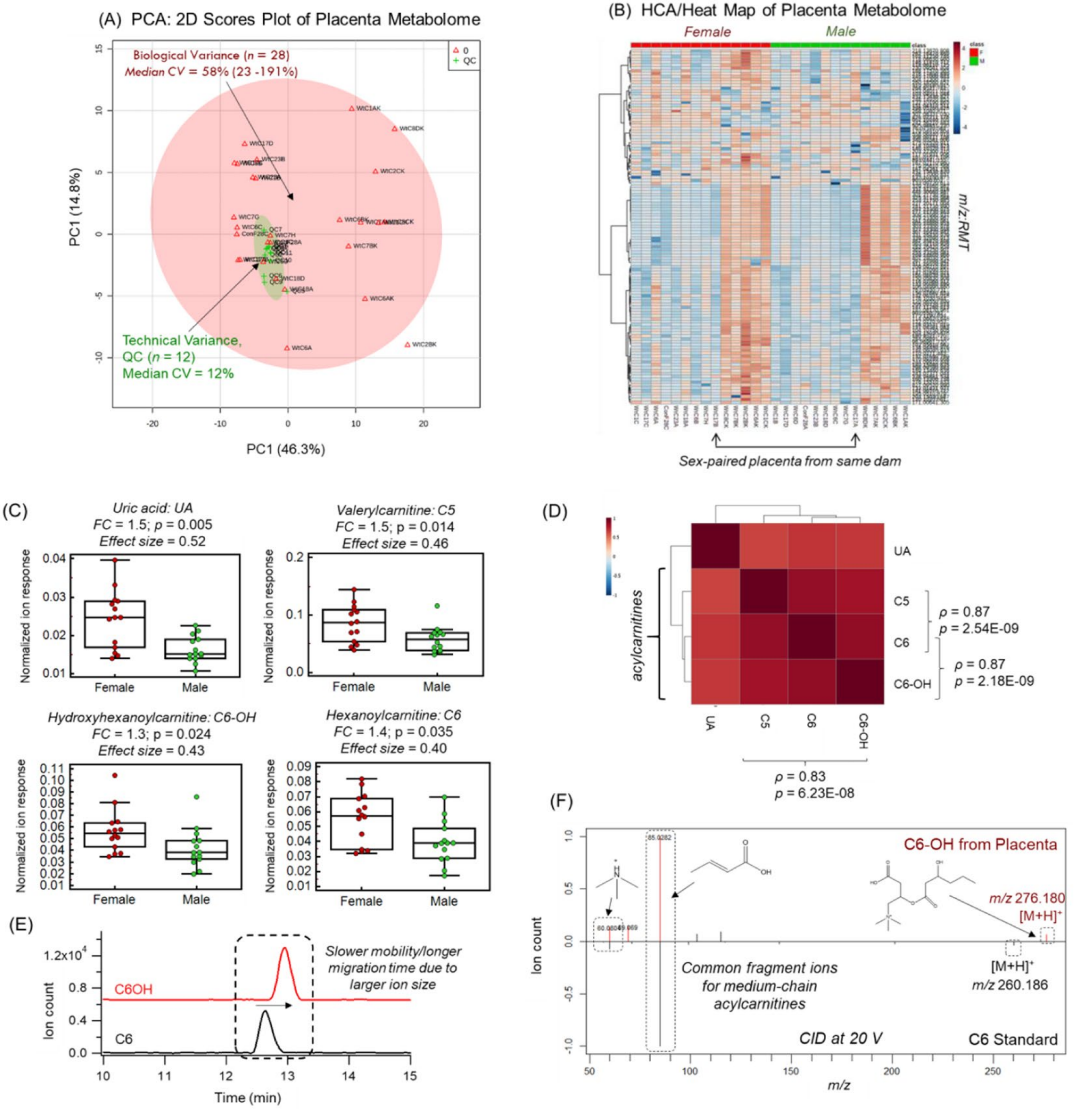
An overview of the murine placental metabolome when using (**A**) 2D PCA scores plot and (**B**) 2D heat map with hierarchical cluster analysis from dams fed a standardized diet prior to and throughout gestation. Metabolite ion responses were normalized to total dried weight (mg), batch-corrected, glog-transformed and autoscaled prior to multivariate statistical analysis. (**C**) Box-whisker plots of top-ranked placental metabolites showing sex-specific differences (*p* < 0.05) based on Mann-Whitney U test. (**D**) Spearman rank correlation matrix of the top-ranked placental metabolites demonstrating strong co-linearity among medium-chain acylcarnitines, C5, C6 and C6-OH (*ρ *> 0.80, *p* < 1.0E-7, *n* = 28). (**E**) Extracted ion electropherograms overlay illustrating a slower positive mobility/longer migration time shift of a unknown ion, tentatively identified as C6-OH as compared to C6 from a pooled placental tissue extract. (**F**) Mirror plot comparing MS/MS spectra acquired with a collision energy at 20 V for an unknown ion, tentatively identified as C6-OH based on comparison to its structural analog, C6. Both spectra depict two common diagnostic product ions at *m/z* 85 and *m/z* 60 consistent with these medium-chain acylcarnitines, whereas their protonated molecular ion [M + H^+^] are offset by m/z 16 due to hydroxyl substituent.

We next aimed to determine sexual dimorphic differences in murine metabolomes from placental extracts derived from paired male (*n* = 14) and female (*n* = 14) fetuses. An overview of the batch-corrected placental metabolome is depicted in Fig. 3(A) when using a 2D scores plot with principal component analysis (PCA) that confirms good technical precision was achieved (median *CV* = 12%, *n* = 12) based on repeated analysis of QCs as compared to the larger biological variation between individual murine placentae (median CV = 58%, *n* = 28) after normalization to total dried weight. A 2D heat map using hierarchical clustering analysis (HCA) is also shown in Fig. 3(B), which highlights the relationship among 122 intracellular metabolites measured between sex-paired placentae from the same dam. Overall metabolic phenotypes between male and female placentae show modest separation when using supervised multivariate analysis based on a partial least squares-discriminate analysis (PLS-DA) as shown in Fig. S2. Also, a variables of importance in projection (VIP ≥ 2.0) lists 6 top-ranked metabolites primarily responsible for sex-dependent differences in murine placental metabolomes, including uric acid, succinic acid, acetylcarnitine and several medium-chain acylarnitines. Univariate statistical analysis was then applied to these top-ranked placental metabolites, which confirmed that 4 compounds were significantly different between female and male placentae following gestation when using Mann-Whitney *U* test as summarized in Table 1. Similarly, Fig. 3(C) depicts box-whisker plots for placental derived uric acid and medium-chain acylcarnitines (*p* < 0.05, effect size >0.40). Importantly, these outcomes were not impacted by the use of the batch correction algorithm when analyzing original data. As expected, there was a strong positive correlation among all 3 medium-chain acylcarnitines (*ρ* > 0.80, *p* < 0.001, *n* = 28) as depicted in the Spearman rank correction matrix in Fig. 3(D). Overall, increases in uric acid (*p* = 0.005), a known purine catabolite, was measured consistently within female placentae as compared to males with similar trends found for medium-chain acylcarnitines, namely valerylcarnitine (*p* = 0.014, C5), hexanoylcarnitine (*p* = 0.035, C6), and an unknown cation (*p* = 0.024, [M + H]^+^). This unknown cation (276.179:0.899:p) was tentatively identified (level 2) as 3-hydroxyhexanoylcarnitine (C6-OH) as shown in Fig. 3(E) based on its characteristic electrophoretic mobility shift as compared to its closest chemical analog commercially available, hexanoylcarnitine (C6) that lacks its substituted hydroxyl group. Also, high resolution MS/MS spectra confirms the detection of two diagnostic product ions (*m/z* 85; *m/z* 60) generated from collision-induced dissociation experiments when comparing C6-OH (from pooled placental extract) with C6 (as an authentic standard) as highlighted in Fig. 3(F). Furthermore, *in silico* fragmentation using MetFragWeb^29^ confirmed excellent spectral matching with experimental MS/MS spectra consistent with a hydroxylated medium-chain acylcarnitine.

**Table 1:** Top-ranked metabolites showing sex-specific differences in female and male placentae following normal murine pregnancies.

*m/z*:RMT:mode	Compound ID	*p*-value^a^	*q*-value^b^	Effect size^c^	FC^d^
167.0211:0.960:n	Uric acid	0.005	0.37	0.52	1.48
246.1700:0.843:p	Valerylcarnitine	0.014	0.58	0.46	1.48
276.1789:0.889:p	Hydroxyhexanoylcarnitine	0.024	0.74	0.43	1.34
260.1856:0.857:p	Hexanoylcarnitine	0.035	0.86	0.40	1.39

^a^Two-tailed exact *p*-values based on Mann-Whitney U test,

^b^*q*-value based on False Discovery Rate (FDR) using Benjamini-Hochberg procedure.

^c^Effect size *r* values estimated from z-scores.

^d^Fold-change (FC) based on the measured ion response ratio of female relative to male placentae.

## Discussion

To date, there have been several metabolomics studies on both human and murine placental tissue samples^16–20^. In most cases however, multiple analytical platforms, including GC-MS, UPLC-MS and/or NMR were needed to achieve adequate coverage due to the chemical diversity of the placental metabolome, which ranges from polar/hydrophilic amino acids to lipophilic long-chain fatty acids. Also, few studies to date have provided reliable quantitative determination of intracellular placental metabolites normalized to dried mass. To the best of our knowledge, this is the first study examining sex-specific metabolic differences on a murine placental model during normal gestation and feeding. MSI-CE-MS offers a high throughput platform for metabolomics with stringent QC that incorporates novel data workflows to facilitate unambiguous authentication of metabolites in murine placental extracts. Importantly, multiplexed separations using a QC-based batch correction algorithm^21,23^ allows for effective adjustment of long-term signal drift in MS-based metabolomics since two independent batches of placental extracts were analyzed intermittently over a 2 year period in this study. A major finding of this work was that there was a 1.5-fold increase in uric acid in female compared to male murine placentae. Uric acid is the terminal end-product of the purine (ATP) degradation pathway, catalyzed by xanthine dehydrogenease (XDH) or xanthine oxidase (XO) in the placenta^30^, which has been associated with deleterious impacts on placental vascular development, structure and function, most notably in cases of preeclampsia^31^. Interestingly, uric acid exhibits antioxidant properties in circulation^32^ by scavenging oxidizing agents (*i.e*., reactive oxygen species, nitric oxide, transition metals) while paradoxically also contributing to oxidative stress and placental inflammation at elevated concentrations (hyperuricemia) upon formation of insoluble uric acid crystals, thereby resulting in fetal growth restriction and adverse pregnancy outcomes^33,34^. Indeed, treatment of pregnant mice with the xanthine oxidase inhibitor, allopurinol, was shown to reduce placental uric acid levels and prevent placental insufficiency due to dietary fructose exposure with improved fetal birth weights^35^. A trend towards higher oxidized to reduced glutathione (GSSG/GSH) ratios and mixed oxidized disulfides (*e.g*., CysGly-CysSS) in female compared to male placentae may also indicate that female placentae are more susceptible to oxidative stress. Therefore elevated uric acid levels within female placentae likely indicates a higher antioxidant capacity to maintain redox homeostasis as compared to males under normal feeding conditions^36^; however, this same metabolic adaptation may become liability to female fetal development when excessive uric acid is generated in response to acute or chronic inflammation during gestation.

Additionally, female placentae may have greater mitochondrial activity as reflected by increased expression of medium-chain acylcarnitines (C5, C6, C6-OH) as compared to males during normal gestation. Carnitine and acylcarnitines play key roles in mitochondrial fatty acid beta-oxidation to fulfill energetic demands for fetal growth and development, especially in later stages of gestation^37^. Previous studies in rodent models^38,39^ have reported sex-dependent differences in intracellular acylcarnitines from other tissues (*i.e*., heart, liver, skeletal muscle); however, the role of fatty acid metabolism remains poorly understood within placenta despite its association with various pathological conditions, including preeclampsia, fetal fatty acid disorders, and maternal liver diseases in pregnancy^40,41^. The higher concentrations of medium-chain acylcarnitines within female placentae may be indicative of fatty acid beta-oxidation as a preferred energetic fuel as compared to male placentae during normal gestation/feeding in the absence of adverse environmental stimuli. For instance, the placentae of obese women have been reported to have fewer mitochondria with lower acylcarnitine concentrations corresponding to reduced fatty acid oxidation capacity than lean women as an adaptive mechanism to restrict excessive fetal adiposity^42^. Similarly, chronic oxidative stress has been shown to impair placental fatty acid oxidation, lipid storage and ATP production leading to pregnancies at risk for fetal growth restriction^43^. Indeed, a modest trend towards increases in acetylcarnitine (*p* = 0.070; effect size = 0.34; *FC* = 1.31) and succinic acid (*p* = 0.094; effect size = 0.32; *FC* = 1.25) in our work highlight consistent trends towards an upregulation in fatty acid and oxidative metabolism in female compared to male placentae. However, there were no sex differences measured in the placental uptake of all major saturated, monounsaturated and polyunsaturated (total hydrolyzed) fatty acids, including essential omega-3 polyunsaturated fatty acids which are important for brain and retinal development^43^, such as DHA (Table S2).

Sexual dimorphism contributes to subtle differences in placental metabolism that likely reflects adaptive responses elicited by the fetus to maximize fitness for optimal growth while ensuring normal development. Evidence of sex specific responses to prenatal nutrition^44–46^, stress^47–50^, hormones and chemicals^51,52^ suggest that the placenta plays a key role in these responses, either through sex chromosomes and/or X inactivation and gene dosage^3,12,53^, or epigenetic mechanisms^1,52,54,55^. In our study, elevated intracellular uric acid measured within female placentae may reflect a counter-balance to compensate for greater oxidative stress, as well as increases in beta-oxidation due to medium-chain acylcarnitine utilization compared to male placentae. While this work provided novel insights into sex-specific metabolic phenotype differences during normal gestation, nontargeted metabolite profiling was limited to placental tissue. Future metabolomics studies that include analysis of maternal blood and fetal cord blood/tissues as complementary biospecimens are needed to better elucidate the interactions between fetus and mother and their impact on placental function, which may also be modulated by dietary exposures during pregnancy^24^. By correlating metabolite changes in the placenta to circulatory measurements in maternal and fetal blood, a more comprehensive understanding of the impact of sexual dimorphism on fetal development may be realized. The integration of other “-omics” approaches with metabolomics can also further validate our findings as related to sex-specific metabolic differences within placenta during fetal development, including the microbiome. Although results from in-bred mice strains may not be equivalent to humans, the development of a mouse placenta is consistent with human placental development as compared to other outbred species, such as rats. Due to the significant biological variance between murine placentae, a larger sample size is needed to further improve study power. Future studies are underway to investigate the impact of maternal nutrition on the long-term health outcomes in offspring, including sex-specific risks for obesity and metabolic syndrome during childhood.

## Methods

### Study design and cohort

This study was approved by the McMaster University Animal Research Ethics Board (Animal Utilization Protocol#12-10-38) and performed in accordance with recommended guidelines and regulations at the McMaster Central Animal Facility. Four week old female C57BL/6J mice (*n* = 14) were fed a standardized diet with a macronutrient composition of 17% kcal fat, 29% kcal protein, and 54% kcal carbohydrate (HT8640 Teklad 22/5 Rodent Diet, Harlan, Indianapolis, IN, USA) for 6 weeks prior to a 5-day mating period with C57BL/6J control-fed male mice. After mating and confirmation of vaginal plug at emboryonic day E18.5, pregnant mice (dams) were killed by cervical dislocation and whole placentae (~7–8 per dam) were dissected without maternal decidua, collected, snap-frozen in liquid nitrogen and stored at −80 °C prior to subsequent lyophilization, liquid extraction and metabolite analysis. Overall, two independent batches of placental specimens from dams were collected and analyzed at different time periods in this study, including a first batch (*n* = 9 female, *n* = 9 male) of placental tissue in 2016, and a second batch (*n* = 5 female, *n* = 5 male) in 2018 in order to further increase study power. It is important to note that murine pregnancies produce multiple fetuses having their own placenta with defined biological sex (*i.e*., placental sex) that is not considered maternal tissue. In this study, we collected one male and one female placenta from each pregnancy (*n* = 14 dams as total sample size) to ensure that an accurate assessment of the biological variability is captured between pregnancies rather than artificially inflating sample size by using the total litter number. As a result, we used only one female and one male fetus from each pregnancy. These placental samples were collected without decidua, which is easily identifiable at E18.5 and was taken in a similar location in the uterus across pregnancies for consistency.

### Sample workup and placental extraction procedure

Murine placental tissues were freeze-dried to form a fine powder to enable accurate weighing using an electronic balance. Lyophilization also enhances extraction efficiency and reproducibility as all ion responses for metabolites were normalized to total dried weight. For the analysis of polar/ionic metabolites, a modified two-step Bligh-Dyer extraction procedure was performed on freeze-dried placental tissue (~2 mg dried tissue) as recently developed for human muscle tissue biospies^23^. Briefly, in the first extraction, 64 µL of ice cold methanol:chloroform (1:1) was added to the tissue, followed by 26 µL ice cold deionized water to induce phase separation. After vortexing for 10 min and centrifugation at 2,000 *g* at 4 °C for 20 min, the upper aqueous layer was aliquoted. A second extraction on the residual placental tissue was performed through the addition of 32 µL of 50% *vol* methanol, followed by vortexing and centrifugation as described above. The second, upper aqueous layer was collected and combined with the first aliquot resulting in ~80 µL total volume of placental tissue extract. Prior to MSI-CE-MS analysis, 5 µL of the internal standards (25 µM), 3-chloro-*L*-tyrosine (Cl-Tyr) and 2-napthalenesulfonic acid (NMS), were added to an aliquot of 20 µL of placenta extract. For the analysis of total (hydrolyzed) fatty acids and bile acids in placental tissue (~1–2 mg dried tissue), a hydrolysis reaction combined with a modified methyl-*tert*-butyl ether (MTBE) extraction procedure was employed^28,56^. First, hydrolysis of lipids was performed by the addition of 25 μL of butylated hydroxytoluene (BHT) in 0.1% *vol* toluene and 25 μL of 2.5 M of sulfuric acid to freeze-dried placental tissue. After vortexing for 1 min, the samples were incubated in an oven at 80 °C for 1 h. Then, a MTBE extraction was performed by adding 500 μL of MTBE containing 50 μM of the stable-isotope labeled recovery standard, myristic acid-d_27_ to placental tissue. After vortexing for 30 min at room temperature, 250 μL of de-ionized water was added to induce phase separation, followed by 30 min of centrifugation at 4,400 *g* at 4 °C. Then, ~250 μL of the upper ether layer was transferred and dried under nitrogen. Prior to MSI-NACE-MS analysis, dried samples were reconstituted in 25 μL of acetonitrile/isopropanol/water (70:20:10) with 10 mM of ammonium acetate and 50 µM of stearic acid-d_35_ as the stable-isotope labeled internal standard used for data normalization.

### Nontargeted metabolomics of placental extracts by MSI-CE-MS

All nontargeted metabolite profiling studies using aqueous and nonaqueous buffer systems were performed on an Agilent G7100 CE System (Agilent Technologies Inc., Mississauga, ON, Canada) coupled to a high resolution Agilent 6230 time-of-flight mass spectrometer (TOF-MS) equipped with a coaxial sheath liquid (Dual AJS) Jetstream electrospray ion source. Separations for polar metabolites were achieved using uncoated fused-silica capillaries (Polymicro Technologies, AZ, USA) with 50 μm inner diameter and 120 cm total length, while 95 cm total length was used for lipid separations. A background electrolyte (BGE) composition of 1 M formic acid with 13% *vol* acetonitrile (pH 1.80) was used for the separation of cationic metabolites, while a BGE comprised of 50 mM ammonium bicarbonate (pH 8.5) was used for anionic metabolite separation. A nonaqueous BGE was used for separations of lipids based on MSI-nonaqueous capillary electrophoresis (NACE)-MS to fully solubilize ionic yet hydrophobic metabolites (*e.g*., fatty acids, bile acids) from ether extracts using 35 mM ammonium acetate (pH 9.5) in 70% *vol* acetonitrile, 15% *vol* methanol, 5% *vol* isopropanol and 10% *vol* de-ionized water. A capillary window maker (MicroSolv, Leland, NC, USA) was used to remove 7 mm of polyimide from the terminal ends to minimize sample carryover and/or capillary swelling upon contact with organic and/or ammonia based buffers^57,58^. The applied voltage was set to 30 kV at 25 °C to enable zonal separations to occur. Moreover, for lipid separations, the Vcap, nozzle voltage and nebulizer gas were turned off during serial sample injection to minimize electrospray suctioning effects. A 7-sample serial injection format was used for nontargeted metabolite profiling of placenta tissue extracts injected hydrodynamically at 50 mbar for 5 s interspaced with seven BGE spacers for 50 s. Therefore, each MSI-CE-MS run consisted of 6 alternating injections of placental tissue extracts that were paired based on female and male placentae collected from the same dam. A pooled quality control (QC) was also included in each experimental run to assess system stability and performance, which was also used to correct for batch effects associated with long-term signal drift during data acquisition. An Agilent 1260 Infinity series isocratic pump equipped with a 100:1 splitter was used to deliver sheath liquid at a rate of 10 μL/min during separations. Sheath liquid compositions consisted of 60% *vol* methanol with 0.1% *vol* formic acid for positive and 50% *vol* methanol for negative ion modes in MSI-CE-MS, whereas 80% *vol* methanol with 0.5% *vol* ammonium hydroxide was the sheath liquid for acidic lipids under negative ion mode when using MSI-NACE-MS. For real-time mass correction during data acquisition, 0.02% *vol* of purine and hexakis (2,2,3,3-tetrafluoropropoxy)phosphazine (HP-921) were added to the sheath liquid. The TOF-MS was performed in full-scan mode over a mass range of *m/z* 50–1700 at an acquisition rate of 500 ms/spectrum. The ESI conditions were Vcap = 2000 V, nozzle voltage = 2000 V, nebulizer gas = 10 psi, sheath gas = 3.5 L/min at 195 °C, drying gas 8 L/min at 300 °C. whereas, the MS voltage settings were fragmentor = 120 V, skimmer = 65 V and Oct1 RF = 750 V. As part of quality assurance practices, the TOF-MS system was calibrated each day using an Agilent tune mixture to ensure mass ranges did not exceed 0.30 ppm. Also, daily cleaning of the CE electrode and ion source with 50% *vol* isopropanol with a lint-free cloth was performed to minimize sample carryover and salt buildup. At the start of each day, a standard metabolite mixture followed by pooled QCs with blank were analyzed to equilibrate the CE-MS system while ensuring good instrumental performance. At the end of each day, the capillary was flushed with 10 min with de-ionized water and air dried for 10 min.

### Tandem mass spectrometry for unknown identification

Tandem mass spectrometry (MS/MS) was utilized in this study for structural elucidation and tentative identification of unknown placental metabolites that significantly differed between sexes. All targeted MS/MS experiments were performed on an Agilent G7100A CE system (Agilent Technologies Inc., Mississauga, ON, Canada) equipped with a coaxial electrospray ionization (ESI) source coupled to an Agilent 6550 iFunnel QTOF-MS. A pooled placenta tissue extract was injected hydrodynamically at 100 bar for 20 s followed by a BGE spacer at 100 mbar for 5 s. Precursor ions were selected for collisional induced dissociation (CID) experiments at 10, 20 and 40 V. The ESI conditions were Vcap = 3500 V, nozzle voltage = 2000 V, nebulizer gas = 8 psi, drying gas 14 L/min at 225 °C, whereas, the MS voltage settings were fragmentor = 380 V and Oct1 RF = 750 V. For structural elucidation, the METLIN database^59^ accessed through the Agilent MassHunter Personal Compound Database and Library (PDCL) manager was used. Since no authentic standards were used to confirm via co-migration with spiking, *in silico* fragmentation using MetFragWeb was employed for MS/MS spectral comparison^29^.

### Data processing and statistical analysis

Data processing was performed using Agilent MassHunter Qualitative Analysis B.06.00 and Microsoft Excel. Prior to statistical analysis, all metabolite responses (relative peak area or RPA) were normalized to the internal standards and the total dried weight of each lyophilized placenta sample (mg) used for extraction. Overall, 135 intra-cellular metabolites were originally detected in this study, however only 122 authenticated metabolites were reliably measured (QC for CV < 30%) in the majority (>75%) of placental extracts. Also, missing data inputs for a given metabolite was substituted with one half of its lowest measured response. Normality testing based on a Shapiro-Wilk test (*p* < 0.05) was performed using SPSS (IBM Corp. Released 2011. IBM SPSS Statistics for Windows, Version 20.0. Armonk, NY: IBM Corp.). A QC-based batch correction algorithm “BatchCorrMetabolomics” R package was used to correct for batch effects^46^. Additional data preprocessing including generalized *log* transformation and autoscaling were performed prior to multivariate statistical analysis. Metaboanalyst 4.0 was used for multivariate statistical analysis including Principal Component Analysis (PCA), Partial Least-Square Discriminant Analysis (PLS-DA) and Hierarchal Clustering Analysis (HCA)^60^. Univariate statistical analysis such as Mann Whitney U test was performed using *log* transformed data (*p* < 0.05) on SPSS for data that was not normally distributed. To correct for multiple hypothesis testing, a false discovery rate (FDR) using the Benjamini-Hochberg procedure was also performed to obtain *q*-values for each top-ranked placental metabolite.

## Conclusion

In summary, this is the first study to apply a high throughput platform for comprehensive metabolite profiling of placental extracts using minimal amounts of freeze-dried tissue (≈1–2 mg). Also, a rigorous data workflow was used for authenticating placental metabolites while implementing stringent quality control measures to minimize false discoveries, including a batch-correction algorithm to correct for long-term signal drift. Over 120 polar/hydrophilic and lipid metabolites were reliably detected by MSI-CE-MS over a wide dynamic range in murine placental tissue extracts when normalized to dried weight. Complementary multivariate and univariate statistical methods revealed differences in placental metabolic profiles among paired male and female placentae from pregnant mice fed a standardized diet six weeks prior to and during gestation with modest effect sizes. Intracellular uric acid and a series of medium-chain acylcarnitines were consistently elevated in female placenta as compared to males, indicative of sex-specific differences in placental fatty acid beta-oxidation activity and antioxidant capacity. Specifically, we demonstrated that female placentae showed a preference towards increased lipid oxidation to fulfill energetic requirements that may require higher uric acid as an antioxidant to maintain redox homeostasis during gestation. Overall, this work provides deeper insights to the subtle role of placental sex on metabolic adaptations that are critical to prevent fetal growth restriction and adverse birth outcomes early in life, including maternal health. Complementary methods for analysis of bioactive lipids and steroid hormones will be developed to further expand coverage of the placental metabolome from mass-limited tissue specimens.

## Supplementary information

Supplemental Information.

Murine Placental Metabolome.

## Data Availability

Supporting tables listing authenticated metabolites identified and quantified from placental extracts using MSI-CE-MS, and supporting figures illustrating batch correction adjustment of long-term signal drift and between-batch data variation, as well as supervised multivariate data analysis of sex-specific differences in placental metabolome using PLS-DA. An excel file [Murine-Placental-Metabolome-SI.xlsx] containing the data matrix of the murine placental metabolome for 122 authenticated metabolites measured by MSI-CE-MS under three different configurations is also provided for full data transparency. All placental metabolites are annotated by their accurate mass and relative migration time (*m/z*:RMT) and name (if identified), where responses reflect their ion response ratio normalized to an internal standard. All sample codes, placental sex, total dried mass used for extraction, and batch number are also listed. This data file is organized into four sheets, including two pairs of uncorrected and batch-corrected placental metabolome data matrices (including QC samples) measured by MSI-CE-MS for polar/ionic metabolites under aqueous buffer conditions, and non-polar/anionic lipids (*i.e*., total hydrolyzed fatty acids).
