# Supplementary material for: Placental Metabolomics for Assessment of Sex-specific Differences in Fetal Development During Normal Gestation
Source: Sci Rep. 2020 Jun 10;10:9399. doi: 10.1038/s41598-020-66222-3 (PMC7286906; doi:10.1038/s41598-020-66222-3)
Supplement: Supplementary file 1 — Supplemental Information. [file 41598_2020_66222_MOESM1_ESM.pdf]

SUPPLEMENTAL INFORMATION

Placental Metabolomics for Assessment of Sex-specific Differences in Fetal Development During Normal Gestation

Michelle Saoi,[1] *Katherine M. Kennedy,*[2] *Wajiha Gohir,*[2] *Deborah M. Sloboda,* [2,3,4]
Philip Britz-McKibbin[1]

[1] Department of Chemistry and Chemical Biology, McMaster University
[2] Department of Biochemistry and Biomedical Sciences, McMaster University
[3] Department of Pediatrics and Obstetrics and Gynecology, McMaster University
[4] Farncombe Family Digestive Health Research Institute, McMaster University

Corresponding author*: Philip Britz-McKibbin (E-mail: britz@mcmaster.ca)

Correspondence: Dr. Philip Britz-McKibbin, Department of Chemistry and Chemical Biology, McMaster University, Hamilton, ON, L8S 4M1, Canada

Fax: +1-905-522-2509

Supplemental Information: Supporting data file description,
Table S1-S2; Figures S1-S2

SUPPLEMENTAL DATA FILE DESCRIPTION

Murine Metabolome Data Matrix by MSI-CE-MS.

An excel file [Murine-Placental-Metabolome-SI.xlsx] containing the data matrix of the murine placental metabolome for 122 authenticated metabolites measured by MSI-CE-MS under three different configurations is also provided for full data transparency. All placental metabolites are annotated by their accurate mass and relative migration time (*m/z*:RMT) and name (if identified), where responses reflect their ion response ratio normalized to an internal standard. All sample codes, placental sex, total dried mass used for extraction, and batch number are also listed. This data file is organized into four sheets, including two pairs of uncorrected and batch-corrected placental metabolome data matrices (including QC samples) measured by MSI-CE-MS for polar/ionic metabolites under aqueous buffer conditions, and non-polar/anionic lipids (*i.e.*, total hydrolyzed fatty acids) under non-aqueous buffer conditions.

Table S1. Comprehensive list of 106 polar metabolites reliably measured in pooled placental tissue extracts by MSI-CE-MS. Metabolites were annotated based on their accurate mass (*m/z*), relative migration time (RMT), mode of ion detection (p or n), most likely molecular formula and mass error. Absolute concentrations for a majority of placental metabolites are also summarized that were normalized to total dried weight.

***m/z*:RMT:mode**	**Compound ID**	**Chemical Formula**	**Mass Error (ppm)**	**Concentration (mmol/kg)**
76.0393:0.731:p	Glycine	$C_2H_5NO_2$	15.3	5.83±0.03
76.0757:0.582:p	Trimethylamine-*N*-oxide	C_3H_9NO	13.3	0.06±0.02
90.0550:0.652:p	β-Alanine	$C_3H_7NO_2$	6.6	--
90.0550:0.782:p	Alanine	$C_3H_7NO_2$	10.1	8.86±0.31
104.0706:0.782:p	γ-Aminobutyric acid*[a]	$C_4H_9NO_2$	1.6	0.053±0.000
104.0706:0.851:p	β-Aminoisobutyric acid*[a]	$C_4H_9NO_2$	0.9	--
104.0706:0.943:p	Dimethylglycine (DMG)	$C_4H_9NO_2$	0.9	0.08±0.01
104.1075:0.605:p	Choline	$C_5H_{14}NO$	1.5	3.13±0.12
106.0499:0.860:p	Serine	$C_3H_7NO_3$	4.1	2.62±0.11
110.0270:1.483:p	Hypotaurine*	$C_2H_7NO_2S$	0.4	--
114.0662:0.648:p	Creatinine	$C_4H_7N_3O$	0.4	0.10±0.04
116.0706:0.917:p	Proline	$C_5H_9NO_2$	1.1	3.14±0.05
118.0611:0.725:p	Guanidoacetic acid*	$C_3H_7N_3O_2$	2.2	--
118.0863:0.852:p	Valine[a]	$C_5H_{11}NO_2$	0.4	1.99±0.45
118.0863:0.960:p	Betaine	$C_5H_{11}NO_2$	6.6	29.6±0.4
120.0655:0.899:p	Threonine	$C_4H_9NO_3$	-2.9	4.83±0.15
120.1020:0.661:p	*Unknown*	$C_5H_{13}NO_2$	0.4	--
122.0270:0.953:p	Cysteine	$C_3H_7NO_2S$	8.0	0.43±0.07
126.0219:1.615:p	Taurine[b]	$C_2H_7NO_3S$	0.2	6.25±0.05
131.1176:0.457:p	*N*-Acetylputrescine*	$C_6H_{14}N_2O$	-5.8	--
132.0655:1.018:p	Hydroxyproline	$C_5H_9NO_3$	2.1	--
132.0768:0.769:p	Creatine	$C_4H_9N_3O_2$	2.1	7.87±0.26
132.1019:0.863:p	Isoleucine[b]	$C_6H_{13}NO_2$	-0.04	--
132.1019:0.873:p	Leucine[b]	$C_6H_{13}NO_2$	-0.04	--
133.0608:0.899:p	Asparagine	$C_4H_8N_2O_3$	-3.8	1.06±0.00
133.0972:0.613:p	Ornithine	$C_5H_{12}N_2O_2$	-8.4	0.11±0.06
134.0448:0.976:p	Aspartic Acid	$C_4H_7NO_4$	0.1	2.88±0.12
137.0458:1.083:p	Hypoxanthine	$C_5H_4N_4O$	1.1	0.37±0.03
137.0709:0.651:p	*N*-Methylnicotinamide*	$C_7H_8N_2O$	-2.7	--

141.0657:0.722:p	*Unknown*	$C_6H_8N_2O_2$	0.9	--
142.0264:1.506:p	*O*-Phosphoethanolamine[b]	$C_2H_8NO_4P$	-1.4	--
146.1181:0.712:p	Deoxycarnitine	$C_7H_{16}NO_2$	-4.4	0.12±0.14
146.1652:0.413:p	Spermidine	$C_7H_{19}N_3$	-1.0	--
147.0764:0.921:p	Glutamine	$C_5H_{10}N_2O_3$	0.3	417.66±0.02
147.1128:0.613:p	Lysine	$C_6H_{14}N_2O_2$	-1.4	3.18±1.66
148.0604:0.933:p	Glutamic Acid	$C_5H_9NO_4$	-0.5	13.0±0.9
150.0583:0.908:p	Methionine	$C_5H_{11}NO_2S$	-1.7	0.92±0.21
151.0431:0.511:p	*Unknown*	$C_5H_{11}O_3S$	-6.2	--
156.0768:0.653:p	Histidine	$C_6H_9N_3O_2$	-0.3	0.64±0.38
160.1330:0.738:p	*Unknown*	$C_8H_{17}NO_2$	0.01	--
162.1125:0.746:p	Carnitine	$C_7H_{15}NO_3$	3.5	12.0±1.2
166.0863:0.933:p	Phenylalanine	$C_9H_{11}NO_2$	-1.5	1.50±0.29
170.0924:0.668:p	3-Methylhistidine	$C_7H_{11}N_3O_2$	-1.0	0.24±0.19
175.1190:0.634:p	Arginine	$C_6H_{14}N_4O_2$	-0.7	0.83±0.16
176.1030:0.944:p	Citrulline	$C_6H_{13}N_3O_3$	-4.5	0.49±0.27
179.0481:0.804:p	Cysteinylglycine*	$C_5H_{10}N_2O_3S$	-1.3	--
182.0812:0.961:p	Tyrosine	$C_9H_{11}NO_3$	-2.4	10.6±0.1
188.1757:0.565:p	*N*-Acetylspermidine*	$C_9H_{21}N_3O$	-0.9	--
189.1346:0.638:p	Homoarginine	$C_7H_{16}N_4O2$	15.3	--
189.1598:0.635:p	Trimethyllysine	$C_9H_{20}N_2O_2$	-15.1	--
203.1503:0.676:p	Asymmetric dimethylarginine	$C_8H_{18}N_4O_2$	3.5	0.04±0.01
203.1503:0.687:p	Symmetric dimethylarginine	$C_8H_{18}N_4O_2$	-1.1	--
203.2230:0.410:p	Spermine	$C_{10}H_{26}N_4$	-4.9	0.16±0.14
204.1230:0.787:p	Acetylcarnitine	$C_9H_{17}NO_4$	1.5	4.13±0.21
205.0972:0.933:p	Tyryptophan	$C_{11}H_{12}N_2O_2$	9.2	366±18
209.0921:0.891:p	Kynurenine	$C_{10}H_{12}N_2O_3$	-4.8	--
218.1387:0.808:p	Propionylcarnitine	$C_{10}H_{19}NO_4$	-0.8	0.05±0.05
230.0958:1.404:p	Ergothioneine*	$C_9H_{15}N_3O_2S$	-0.03	--
232.1543:0.820:p	Isobutyrylcarnitine[a]	$C_{11}H_{21}NO_4$	-0.01	--
232.1543:0.827:p	Butyrylcarnitine[a]	$C_{11}H_{21}NO_4$	-0.01	0.04±0.02
242.5621:0.883:p	CysGly-GSH-SS*	$C_{15}H_{25}N_5O_9S_2$	-1.2	--
243.0737:1.044:p	*Unknown*	$C_9H_6N_8O$	-2.9	--
244.0991:0.848:p	*Unknown*	$C_{11}H_{17}NO_3S$	2.7	--

246.1700:0.837:p	Isovalerylcarnitine[a]	$C_{12}H_{24}NO_4$	-1.1	--
246.1700:0.843:p	Valerylcarnitine[a]	$C_{12}H_{24}NO_4$	-1.4	--
248.1492:0.852:p	Hydroxybutyrylcarnitine	$C_{11}H_{21}NO_5$	-0.2	--
258.1097:1.592:p	Glycerophosphoylcholine*[b]	$C_8H_{20}NO_6P$	-0.1	--
260.1856:0.857:p	Hexanoylcarnitine	$C_{13}H_{25}NO_4$	0.4	--
262.1647:0.869:p	*Unknown*	$C_{12}N_{23}NO_5$	-1.3	--
268.1040:0.871:p	Adenosine	$C_{10}H_{13}N_5O_4$	-1.9	--
269.0878:1.528:p	Inosine[b]	$C_{10}H_{12}N_4O_5$	-1.0	--
276.1551:0.833:p	*Unknown*	$C_{12}H_{17}N_7O$	8.1	--
276.1789:0.889:p	Hydroxyhexanoycarnitine	$C_{13}H_{25}NO_5$	-5.7	--
284.0989:1.149:p	Guanosine	$C_{10}H_{13}N_5O_5$	-3.2	--
298.0526:0.828:p	CysGly-Cys-SS*	$C_8H_{15}N_3O_5S_2$	39.5	--
298.0959:0.883:p	5-Methylthioadenosine*	$C_{11}H_{15}N_5O_3S$	-4.4	--
307.0833:1.030:p	Oxidized glutathione (GSSG)	$C_{20}H_{32}N_6O_{12}S_2$	0.9	1.40±0.46
308.0911:1.108:p	Reduced glutathione (GSH)	$C_{10}H_{17}N_3O_6S$	0.3	4.87±0.71
427.0952:0.990:p	Cys-GSH-SS	$C_{13}H_{22}N_4O_8S_2$	-1.0	0.23±0.11
89.0244:1.155:n	Lactic acid	$C_3H_6O_3$	7.3	18.9±1.8
96.9696:1.720:n	Inoganic phosphate	H_3O_4P	0.2	12.6±0.1
103.0401:1.023:n	3-Hydroxybutyric acid	$C_4H_8O_3$	-0.01	3.77±2.74
103.0401:1.048:n	2-Hydroxybutyric acid	$C_4H_8O_3$	-0.3	0.11±0.02
117.0193:1.906:n	Succinic acid	$C_4H_6O_4$	1.4	3.27±1.84
128.0353:1.019:n	Oxoproline	$C_5H_7NO_3$	-8.7	0.38±0.04
133.0142:1.927:n	Malic acid	$C_4H_6O_5$	-1.1	--
167.0211:0.960:n	Uric acid	$C_5H_4N_4O_3$	4.4	0.88±0.02
171.0064:1.305:n	Glycerol 3-phosphate	$C_3H_9O_6P$	9.6	--
175.0252:0.911:n	Ascrobic Acid*	$C_6H_8O_6$	2.1	--
179.0561:0.568:n	Glucose	$C_6H_{12}O_6$	19.3	--
180.9520:1.720:n	*Unknown*	$C_6H_2N_2OS_2$	1.8	--
187.0426:1.155:n	*Unknown*	$C_8H_{12}O_3S$	0.9	--
191.0197:2.106:n	Citric acid/Isocitric acid	$C_6H_8O_7$	-6.6	--
195.0510:0.879:n	Gluconic acid	$C_6H_{12}O_7$	14.7	--
218.1034:0.823:n	Pantothenic acid	$C_9H_{17}NO_5$	0.9	0.05±0.01
259.0224:1.058:n	Glucose-6-phosphate[b]	$C_6H_{13}O_9P$	-6.6	0.39±0.01
264.9520:1.782:n	1,3-Bisphosphoglycerate*	$C_3H_8O_{10}P_2$	1.7	--

338.9888:1.534:n	Fructose-1,6-bisphosphate	$C_6H_{14}O_{12}P_2$	-5.3	0.62±0.07
346.0558:1.006:n	AMP	$C_{10}H_{14}N_5O_7P$	-2.2	1.27±0.08
347.0398:1.027:n	IMP[b]	$C_{10}H_{13}N_4O_8P$	-8.6	0.31±0.04
426.0221:1.176:n	ADP	$C_{10}H_{15}N_5O_{10}P_2$	0.0	0.33±0.03
482.9613:1.306:n	UTP[b]	$C_9H_{15}N_2O_{15}P_3$	-20.6	0.60±0.38
505.9885:1.220:n	ATP	$C_{10}H_{16}N_5O_{13}P_3$	-1.3	1.44±0.44
521.9834:1.196:n	GTP[b]	$C_{10}H_{16}N_5O_{14}P_3$	0.03	1.02±0.77
558.0644:0.916:n	ADP-Ribose*	$C_{15}H_{23}N_5O_{14}P_2$	0.2	--
662.1024:0.705:n	NAD^+	$C_{21}H_{28}N_7O_{14}P_2$	-5.7	0.70±0.04

[a] *Suffer from isobaric/isomeric interference using MSI-CE-MS analysis,* [b] *Not included in final study matrix, *tentatively identified based on MS/MS spectral matching to public databases*

Table S2. Summary of 29 hydrolyzed (total) fatty acids and other acidic lipids reliably measured in placental tissue extracts by MSI-NACE-MS. Metabolites are annotated based on their accurate mass (*m/z*), relative migration time (RMT), mode of ion detection (p or n), most likely molecular formula and mass error. Absolute concentrations for placental derived fatty acids are also summarized that were normalized to total dried weight.

***m/z*:RMT:mode**	**Compound ID**	**Chemical Formula**	**Mass Error (ppm)**	**Concentration (mmol/kg)**
199.1703:1.032:n	Lauric acid (12:0)[b]	$C_{12}H_{24}O_2$	-2.8	0.13±0.09
227.2017:1.004:n	Myristic acid (14:0)	$C_{14}H_{28}O_2$	-3.0	0.44±0.15
241.2173:0.990:n	Pentadecanoic acid (15:0)	$C_{15}H_{30}O_2$	-2.4	0.33±0.08
253.2173:0.985:n	Palmitoleic acid (16:1)	$C_{16}H_{30}O_2$	-1.7	1.54±0.47
255.2329:0.981:n	Palmitic acid (16:0)	$C_{16}H_{32}O_2$	4.3	20±2
267.2330:0.975:n	Heptadecenoic acid (17:1)	$C_{17}H_{32}O_2$	-0.8	
269.2486:0.966:n	Heptadecanoic acid (17:0)	$C_{17}H_{34}O_2$	-2.1	0.104±0.067
277.2173:0.983:n	γ-Linolenic acid (18:3)[a]	$C_{18}H_{30}O_2$	-2.9	
277.2173:0.981:n	α-Linolenic acid (18:3)[a]	$C_{18}H_{30}O_2$	-2.3	
279.2330:0.967:n	Linoleic acid (18:2)	$C_{18}H_{32}O_2$	0.4	8.69±2.28
281.2486:0.961:n	Oleic acid (18:1)	$C_{18}H_{34}O_2$	0.8	9.08±2.29
283.2642:0.954:n	Stearic acid (18:0)	$C_{18}H_{36}O_2$	2.6	48±4
297.2799:0.942:n	Nonadecanoic acid (19:0)	$C_{19}H_{38}O_2$	-20.5	
301.2173:0.981:n	Eicosapentaenoic acid (20:5*n*-3)	$C_{20}H_{30}O_2$	59.5	0.88±0.28
303.2330:0.978:n	Arachidonic acid (20:4*n*-6)	$C_{20}H_{32}O_2$	2.4	12±3
305.2486:0.953:n	Eicosatrienoic acid (20:3)	$C_{20}H_{34}O_2$	-0.3	
307.2642:0.942:n	Eicosadienoic acid (20:2)	$C_{20}H_{36}O_2$	-3.6	
309.2799:0.937:n	Eicosenoic acid (20:1)	$C_{20}H_{38}O_2$	-6.7	
311.2955:0.931:n	Arachidic acid (20:0)	$C_{20}H_{40}O_2$	-3.4	
327.2330:0.988:n	Docosahexaenoic acid (22:6*n*-3)	$C_{22}H_{32}O_2$	1.3	6±1
329.2486:0.982:n	Docosapentaenoic acid (22:5)	$C_{22}H_{34}O_2$	11.8	
331.2643:0.939:n	Docosatetraenoic acid (22:4)	$C_{22}H_{36}O_2$	-0.3	
337.3112:0.918:n	Erucic acid (22:1)	$C_{22}H_{42}O_2$	10.9	
339.3269:0.913:n	Docosanoic acid (22:0)	$C_{22}H_{44}O_2$	-0.8	
365.3425:0.901:n	Nervonic acid (24:1)	$C_{24}H_{46}O_2$	-3.2	
367.3582:0.898:n	Tetracosanoic acid (C24:0)	$C_{24}H_{48}O_2$	-3.0	
407.2803:0.951:n	Cholic acid	$C_{24}H_{40}O_5$	7.5	
448.3068:0.987:n	Glycochenodeoxycholic acid	$C_{26}H_{43}NO_5$	-63.9	
565.5211:0.958:n	*Unknown*	$C_{36}H_{69}O_4$	-9.4	

[a] *Isomeric FA were not fully resolved by MSI-CE-MS analysis;* [b] *Not included in final study matrix*

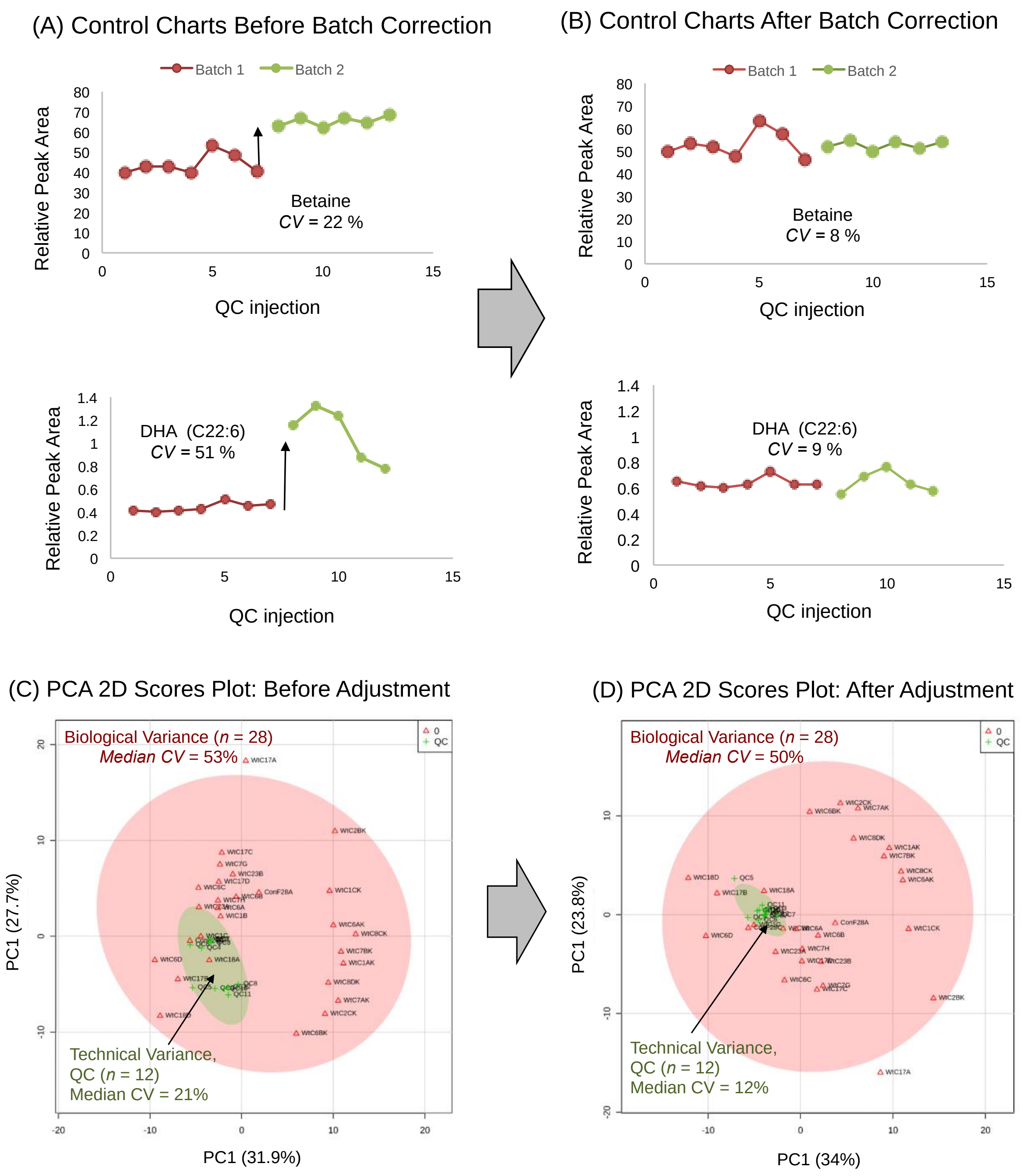

Figure S1. Control charts of placental QCs of betaine (118.086:0.960:p) and docosahexaenoic acid, DHA (327.233:0.988:n) before **A** and after **B** batch correction was used to correct for signal drift in ESI-MS to improve long-term technical precision when both analytical batches were combined. The red lines represent batch 1 analyzed in 2016, while the green lines represent batch 2 analyzed in 2018. 2D scores plots from PCA **C** before and **D** after application of QC based batch-correction algorithm, where overall technical variance significantly decreased from 21% to 12% without changing natural biological variance based on 122 metabolites reliably measured in the placental QCs (*n*=12).

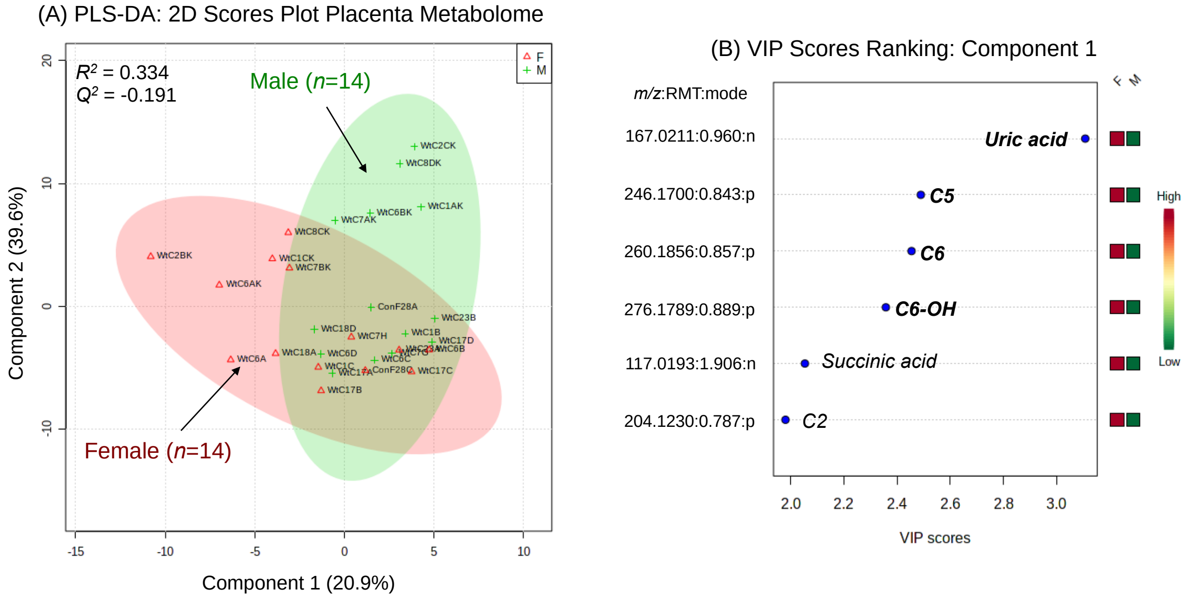

Figure S2. A A 2D PLS-DA scores plot showing sex-dependent differences in the murine placental metabolome following normal gestation and a standardized diet. Metabolite ion responses were normalized to an internal standard and placental dried weight (mg), and then autoscaled and batch-corrected following a perturbation test. **B** Variance in Projection (VIP) plot along component 1 which lists the top-ranked metabolites discriminating between sex from placentae along the first component. Bolded metabolites are significant ($p < 0.05$) when using a Mann Whitney U test. C5: valerylcarnitine, C6: hexanoylcarnitine, C6-OH: 3-hydroxyhexanoylcarnitine.
